# UNRAVELING NONGENOMIC MECHANISMS BY WHICH 17A-ESTRADIOL EXTENDS HEALTHSPAN AND LONGEVITY

**DOI:** 10.1093/geroni/igad104.2497

**Published:** 2023-12-21

**Authors:** Helena Zomer, Vijay Sirohi, Theresa Medrano, Paul Cooke

**Affiliations:** University of Florida, Gainesville, Florida, United States; University of Florida, Gainesville, Florida, United States; University of Florida, Gainesville, Florida, United States; University of Florida, Gainesville, Florida, United States

## Abstract

Recent work indicates that exogenous 17α-estradiol (17α-E2), a stereoisomer of 17β-E2, produces unprecedented longevity increases (~20%) in male mice, challenging prevailing assumptions that 17α-E2 is a weak estrogen. However, is unclear how 17α-E2 acts. Therefore, this study aimed to obtain mechanistic insights into 17α-E2 actions in the uterus, the most widely used vivo model system for estrogen action. We hypothesized that 17α-E2 signals through membrane (m)ESR1 to affect target tissues and promote longevity. To test this, we treated pre-pubertal (postnatal day 20) female CD-1 mice with 3 daily IP injections of vehicle or 10 mg/kg of 17α-E2 or 17β-E2 (n=4/group). 17α-E2 treatment did not significantly increase uterine weight – a marker of nuclear (n)ESR1 signaling (20.3 ± 2.1 mg vs. vehicle 15.7 ± 2.0 mg, p>0.05), while 17β-E2 strongly increased it to 41.4 ± 1.1 mg (p< 0.05). Accordingly, histological analysis revealed reduced cell proliferation in 17α-E2- vs. 17β-E2-treated uteri. Both 17α-E2 and 17β-E2 robustly stimulated epithelial p-ERK and p-AKT expression, the active phosphorylated forms of these protein kinases, and western blotting showed a strong and comparable stimulation of EZH2, a critical mediator of estrogen’s epigenetic effects, which are linked to mESR1 signaling. In conclusion, our results indicate that despite limited actions through nESR1, 17α-E2 robustly signals through mESR1 to activate uterine protein kinase cascades and produce epigenetic effects. These findings may cause a significant reappraisal of 17α-E2 biological effects and role in normal estrogen physiology and provide critical insights into its unique ability to stimulate longevity.

